# Tumor burden and location as prognostic factors in patients treated by iodine seed implant brachytherapy for localized prostate cancers

**DOI:** 10.1186/s13014-019-1449-z

**Published:** 2019-12-31

**Authors:** Claire Meynard, Andres Huertas, Charles Dariane, Sandra Toublanc, Quentin Dubourg, Saik Urien, Marc-Olivier Timsit, Arnaud Méjean, Nicolas Thiounn, Philippe Giraud

**Affiliations:** 1 Hôpital Européen Georges Pompidou, 20 rue Leblanc, 75015 Paris, France; 2 Unité de Recherche Clinique, Hôpital Tarnier, 89 rue d’Assas, 75006 Paris, France

**Keywords:** Prostate cancer, Iodine seed implant brachytherapy, Prostate biopsies, Tumor burden, Gleason score, Risk groups

## Abstract

**Background:**

Iodine seed implant brachytherapy is indicated for low risk and selected favorable intermediate risk prostate cancers. A percentage of positive biopsies > 50% is usually considered as a contra-indication, and the tumor location could also influence the treatment efficacy. We studied the association of the percentage of positive biopsy cores, and tumor location, with progression-free survival.

**Methods:**

Among the 382 patients treated at our center by permanent implant iodine seed brachytherapy for a localized prostate cancer between 2006 and 2013, 282 had accessible detailed pathology reports, a minimum follow-up of 6 months, and were included. Progression was defined as a biochemical, local, nodal, or distant metastatic relapse. We studied cancer location on biopsies (base, midgland or apex of the prostate) and percentage of positive biopsy cores, as well as potential confounders (pre-treatment PSA, tumor stage, Gleason score, risk group according to D’Amico’s classification modified by Zumsteg, adjunction of androgen deprivation therapy, and dosimetric data).

**Results:**

Most patients (197; 69.9%) had a low risk, 67 (23.8%) a favorable intermediate risk, 16 (5.7%) an unfavorable intermediate risk, and 1 (0.3%) a high-risk prostate cancer. An involvement of the apex was found for 131 patients (46,5%), of the midgland for 149 (52,8%), and of the base for 145 (51,4%). The median percentage of positive biopsy cores was 17% [3–75%]. The median follow-up was 64 months [12–140]. Twenty patients (7%) progressed: 4 progressions (20%) were biochemical only, 7 (35%) were prostatic or seminal, 6 (30%) were nodal, and 3 (15%) were metastatic. The median time to failure was 39.5 months [9–108]. There were more Gleason scores ≥7 among patients who progressed (40% vs 19%; *p* = 0.042). None of the studied covariates (including tumor location, and percentage of positive biopsy cores), were significantly associated with progression-free survival. The risk group showed a trend towards an association (*p* = 0.055).

**Conclusions:**

Brachytherapy is an efficient treatment (5-year control rate of 93%) for patients carefully selected with classical criteria. The percentage and location of positive biopsies were not significantly associated with progression-free survival. A Gleason score ≥ 7 was more frequent in case of progression.

## Background

Low-dose rate prostate brachytherapy, with permanent implant of Iodine-125 seeds, is an effective treatment for patients with low to intermediate-risk localized prostate cancer [1–4]. The safety profile of brachytherapy makes it an interesting alternative to radical prostatectomy and external beam radiation therapy [5, 6], and its good results in terms of oncological outcome [7–10] make it an attractive alternative to active surveillance too, for patients refusing it.

Brachytherapy is recommended for patients with low-risk prostate cancer and selected favorable intermediate risk prostate cancer (with only one factor of intermediate risk among a Gleason score of 7(3 + 4) and a pre-treatment PSA serum level between 10 and 15 ng/mL) [11, 12]. Patients should also have an International Prostatic Symptom Score (IPSS) of 12 or less, and a prostatic volume of less than 50 cc [11].

Some guidelines include a threshold on the percentage of positive prostate biopsy cores, which should not exceed 50% [13]. It is even restricted to 33% for patients with a Gleason score of 7, according to other guidelines [14]. The percentage of positive biopsies has been shown to be associated with biochemical outcome after prostatectomy, and after external-beam radiation therapy [15]. Concerning brachytherapy, although some retrospective studies showed a relationship between the percentage of positive biopsies and the biochemical progression free survival [16–20], this threshold of 50% of positive prostate biopsies is not commonly admitted.

Another limitation that is sometimes considered when treating a prostate cancer with brachytherapy, is the location of the cancer in the prostate gland. Classically, the prostate gland is divided into six sextants, by splitting it into left and right, and into three equal segments along the longest sagittal dimension (base, midgland, and apex).

The appropriateness of brachytherapy for cancers involving the base of the prostate has been questioned [21]. The prostate base has indeed been shown to be less well covered with brachytherapy [22]. There are several explanations for this underdosage of the prostate base. First is the proximity of the bladder neck, which must be spared to avoid urinary toxicity [23]. Second is the difficulty in contouring the prostate base with ultra-sound images [24]. Additionally, seed location is more hazardous in the prostate base, due to greater seed displacements [25], loss in the voided urines and lung embolization [26], and because of needle splay (needle divergence, affecting mostly the distribution of superior sources) and needle drag (sources dragged by the needles when pulled back, resulting in a position that is inferior to the initially intended coordinates) [26, 27].

This study aims at reporting the importance of prostate cancer location, from diagnostic prostate biopsies, and percentage of positive biopsy cores, in predicting relapse for patients treated with low-dose rate brachytherapy.

## Methods

### Patients

We retrospectively reviewed the medical records of all patients who were treated at the radiotherapy department of the European Hospital Georges Pompidou with permanent low-dose Iodine seed implant brachytherapy for a newly diagnosed localized prostate cancer between 2006 and 2013.

Patients with available detailed pre-treatment pathology reports and a minimum follow-up of 6 months were included. Written consent was obtained from all patients.

All patients were classified, according to the D’Amico classification [28], into low-risk disease (clinical tumor stage ≤ T2a, PSA serum level < 10 ng/mL, and Gleason score ≤ 6), intermediate-risk disease (clinical stage T2b, PSA between 10 and 20 ng/mL, or Gleason score of 7 (3 + 4 or 4 + 3)), or high risk-disease (clinical stage ≥ T2c, PSA > 20 ng/mL, or Gleason ≥8). The intermediate risk disease category was subclassified, according to Zumsteg [29], into favorable intermediate risk disease (only one factor of intermediate risk disease, Gleason ≤7(3 + 4), and percentage of positive biopsy cores < 50%), and unfavorable intermediate risk disease (intermediate risk disease that cannot be classified as favorable intermediate risk disease). Another risk group classification was studied, using the same criteria except for the tumor stage, which was radiological instead of clinical.

### Patient evaluation

For all patients, the prostate cancer had been clinically staged using medical history, physical examination (including digital rectal examination), and serum PSA measurements. Urinary function was evaluated with the IPSS questionnaire and erectile and bowel functions were assessed during patient interview. A prostate MRI was realized for all patients in our cohort but one.

Pathologic confirmation of the diagnosis of prostate adenocarcinoma was obtained for all patients with ultrasound guided biopsies.

### Treatment

All patients received permanent low-dose Iodine seed implant brachytherapy with a curative intent. Treatment planning was done at the time of the procedure, with Variseed planning software (Varian medical systems, Palo Alto, CA). The prescription dose was 160 Gy. The treatment volume included the whole prostate. The implantation procedure was performed with 18-gauge needles by a radiation oncologist and a urologist, in collaboration. Delineation of the prostate, needle insertion and seed placement were performed under trans-rectal sonographic guidance using a transperineal template. Needles were loaded manually with loose seeds containing permanent Iodine-125 sources (with an activity of 0.4935 mCi/seed). Upon completion of the procedure, patients underwent an immediate anterior X-ray. Prostate coverage was also evaluated more precisely with a CT-scan (immediately and at one month for patients treated before 2018, then at 2 months only).

Androgen deprivation therapy was offered before brachytherapy for cytoreductive purpose at physician’s discretion.

The dosimetric parameters evaluated included the D90 (the minimum dose received by 90% of the volume), the V100 (percentage of the volume receiving 100% of the prescribed dose) and the V150 (percentage of the volume receiving 150% of the prescribed dose) for the target volume; the D10 (the minimum dose received by 10% of the volume) and D30 (the minimum dose received by 30% of the volume) for the urethra. Unfortunately, the sector analysis by sextant for these dosimetric parameters was not available.

### Follow-up

Patients were monitored with physical examination and PSA level measurements every 6 months for 5 years, then every year. After ten years of follow-up with no evidence of relapse, patients were usually referred to their general practitioner for the pursuit of the follow-up.

The biochemical relapse was defined as an elevation of the PSA level of at least 2 ng/mL above the nadir of the PSA levels, according to the Phoenix definition [30].

Treatment failure was defined as biochemical relapse, positive biopsy findings, or radiographic evidence of local disease progression or distant metastases. Failures were classified into biochemical relapse, local prostatic relapse or relapse in the seminal vesicles, pelvic nodal relapse, extra-pelvic nodal relapse, and metastatic relapse.

Patients who showed no evidence of treatment failure at the time of our analysis were classified as disease free.

### Statistics

The association between progression free survival and each of the following variables was studied, using a Cox regression model: percentage of positive biopsies, location of positive biopsies (studying the apex involvement, base involvement, and median involvement), pre-treatment PSA serum level, Gleason score, clinical and radiological tumoral stage, clinical risk group, radiological risk group, adjunction of cytoreductive androgen deprivation therapy and its duration, D90 and V100. A Kaplan-Meier analysis was performed to generate progression free survival curves. The correlation between tumor location on MRI and on biopsies was tested using Pearson’s chi squared test and Cohen’s kappa statistic [31].

Statistical analysis was performed using R software (R foundation for Statistical Computing, Vienna, Austria) [32–34].

## Results

Of the 382 patients who were treated at our center between 2006 and 2013, 282 had detailed pre-treatment pathology reports and a minimum follow-up of 6 months, and were included.

The characteristics of the patients are presented in Table 1. Among the 282 patients, most patients (69.9%) presented with a low-risk disease, according to the clinical D’Amico classification, modified by Zumsteg. The Gleason score was 6 (3 + 3) for 213 patients (75.5%), 7(3 + 4) for 52 patients (18.4%), and 7(4 + 3) for eight patients (2.8%). The median pre- treatment PSA serum level was 6.4 ng/mL (ranging from 0.9 to 17 ng/mL).

**Table 1:** Population characteristics

Characteristics	Total population (*n* = 282)	Progression group (*n* = 20)	Progression-free group (*n* = 262)
Clinical risk group	Low: 197 (69.8%)	Low: 9 (45%)	Low: 188 (71%)
	Intermediate: 83 (29.4%)	Intermediate: 10 (50%)	Intermediate: 73 (27.9%)
	Favorable intermediate: 67 (23%)	Favorable intermediate: 8 (40%)	Favorable intermediate: 59 (22%)
	Unfavorable intermediate: 16 (5.6%)	Unfavorable intermediate: 2 (10%)	Unfavorable intermediate: 14 (5%)
	High: 1 (0.3%)	High: 0	High: 1 (0.3%)
	NA: 1	NA: 1	
Radiological risk group	Low: 158 (56%)	Low: 9 (45%)	Low: 150 (57.3%)
	Intermediate: 84 (29.7%)	Intermediate: 10 (50%)	Intermediate: 75 (28.6%)
	Favorable intermediate: 67 (23.7%)	Favorable intermediate: 8 (40%)	Favorable intermediate: 60 (22.9%)
	Unfavorable intermediate: 17 (6%)	Unfavorable intermediate: 2 (10%)	Unfavorable intermediate: 15 (5.7%)
	High: 33 (11.7%)	High: 0	High: 31 (11.8%)
	NA: 7	NA: 1	NA: 6
Clinical Tumor stage	T1c: 226 (80%)	T1c: 15 (75%)	T1c: 211 (80.5%)
	T2a: 50 (17.7%)	T2a: 3 (15%)	T2a: 47 (17.9%)
	T2b: 4 (1.4%)	T2b: 1 (5%)	T2b: 3 (1.1%)
	T2c: 1 (0.3%)	T2c: 0	T2c: 1 (0.3%)
	NA: 1	NA: 1	
Radiological Tumor stage	T1c: 98 (34.7%)	T1c: 5 (25%)	T1c: 93 (35.5%)
	T2a: 124 (44%)	T2a: 10 (50%)	T2a: 114 (43.5%)
	T2b: 20 (7.1%)	T2b: 2 (10%)	T2b: 18 (6.9%)
	T2c: 32 (11.3%)	T2c: 2 (10%)	T2c: 30 (11.5%)
	T 3a: 1 (0.3%)	T 3a: 0	T3a: 1 (0.3%)
	NA: 7	NA: 1	NA: 6
Gleason score	4: 2 (0.7%)	4: 0	4: 2 (0.8%)
	5: 7 (2.5%)	5: 1 (5%)	5: 6 (2.3%)
	6: 213 (75.5%)	6: 11 (55%)	6: 202 (77.1%)
	7 (3 + 4): 52 (18.4%)	7 (3 + 4): 7 (35%)	7 (3 + 4): 45 (17.2%)
	7 (4 + 3): 8 (2.8%)	7 (4 + 3): 1 (5%)	7 (4 + 3): 7 (2.7%)
Pre-treatment PSA serum level	6.4 ng/mL [0.9–17]	6.6 [3.76–11.9]	6.4 [0.9–17]
Cytoreductive hormone therapy	Yes: 131 (46.4%)	Yes: 12 (60%)	Yes: 119 (45.4%)
	No: 148 (52.4%)	No: 8 (40%)	No: 140 (53.4%)
	NA: 3		NA: 3
Duration of cytoreductive hormone therapy	3 months [0.5–12]	2 months [1–8]	3 months [0.5–12]
Location of positive biopsy cores	Base: 145 (51.4%)	Base: 11 (55%)	Base: 134 (51.1%)
	Midgland: 149 (52.8%)	Midgland: 10 (50%)	Midgland: 139 (53.1%)
	Apex: 131 (46.5%)	Apex: 10 (50%)	Apex: 121 (46.2%)
	NA: 37	NA: 3	NA: 34
Percentage of positive biopsy cores	Median: 17%	Median: 17%	Median: 17%
	< 50%: 262 (92.3%)	< 50%: 17 (92.3%)	< 50%: 245 (93.5%)
	≥ 50%: 14 (4.9%)	≥ 50%: 3 (4.9%)	≥ 50%: 11 (4.2%)
	NA: 6	NA: 0	NA: 6
Radiological location of tumor	None: 90 (31.9%)	None: 5 (25%)	None: 85 (32.4%)
	Base: 72 (25.5%)	Base: 8 (40%)	Base: 64 (24.4%)
	Midgland: 112 (39.7%)	Midgland: 11 (55%)	Midgland: 101 (38.5%)
	Apex: 63 (22.3%)	Apex: 6 (30%)	Apex: 57 (21.8%)
	NA: 16	NA: 1	NA: 15
Disease relapse	Yes: 20 (7.1%)		
	No: 262 (92.9%)		
Death	Yes: 2 (0.7%)	Yes: 0	Yes: 2 (0.7%)
	No: 280 (99%),	No: 20	No: 260 (99%)
Median D90 (Gy)	181 [153–189]	181 [171–189]	181 [153–189]
Median V100 (%)	100 [99–100]	100 [99,7–100]	100 [99–100]
Median V150 (%)	49.2 [31.5–64.8]	49.2 [33–55]	49.2[31–65]
Median D10% urethra	196.5 [168.6–240]	200 [182–226]	196 [168–240]
Median D30% urethra	183[102.9–212.2]	183 [165–205]	182.9 [102–212]

Abbreviations: *NA* Not available

The median number of prostate biopsies that were performed was 12 (ranging from 5 to 32). Targeted biopsies were realized for 28 patients (9.9%). A total of 149 patients (52.8%) had a disease involving the midgland; 131 (46.5%) involving the apex, and 145 (51.4%) involving the prostate base. The location was not detailed for 37 patients (13.1%). Concerning percentage of positive biopsy cores, 262 patients (92.3%) had less than 50% positive biopsy cores, and 14 (4.9%) had 50% or more positive biopsies. The information was not available for 6 patients (2.1%). The median percentage of positive biopsy cores was 17% [3–75%].

The prostatic volume evaluated initially on MRI, was available for 240 patients. The median value was 35 cm^3^ (ranging from 12 to 100 cc). The prostatic volume evaluated on the day of the procedure, with ultrasound imaging, was available for 203 patients, with a median value of 29.8 cc (ranging from 10.5 to 62.2 cc).

One hundred and thirty-one patients (46%) received androgen deprivation therapy before brachytherapy for cytoreduction purposes. The duration of the androgen deprivation therapy was 3 months in median (ranging from 0.5 to 12 months) for these patients. No patient received adjuvant androgen deprivation therapy.

The correlation between tumor location according to the MRI and tumor location according to the prostate biopsies was poor (with a Pearson’s chi squared test *p*-value of 0.0033, and a Cohen’s kappa statistic of 0.1).

**Fig. 1:**
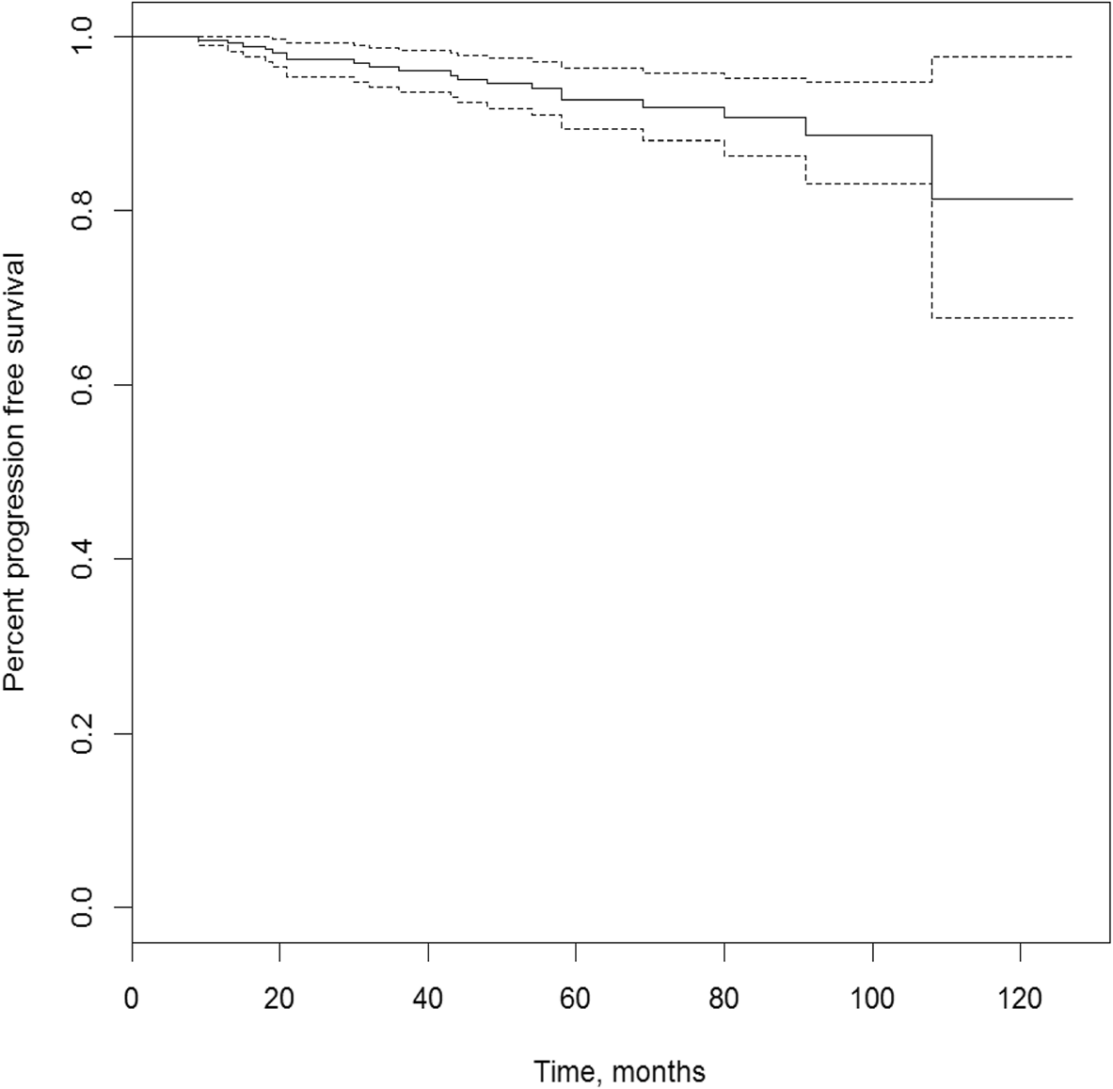
Progression-free survival rate

The median follow-up for the vital status was 64 months [12–140], and 61 months [5–137] for the progression status. The five-year progression free survival was 92.8% (95% confidence interval [89.3–96.3%]) (Fig. 1). Twenty patients presented a progression, four were biochemical progressions only, seven were intra prostatic or seminal failures, five were pelvic nodal failures, one was an extra-pelvic nodal failure, and three were metastatic failures. The median time to progression was 39,5 months (ranging from 9 to 108 months). Two patients died, of causes unrelated to their prostate cancer (one from glioblastoma and one from ethylic cirrhosis). The characteristics of the relapse for the seven patients who presented an intra-prostatic or seminal failure are presented in Table 2.

**Table 2:** Characteristics of the local relapses

	Initial tumor location on biopsies	Initial tumor location on MRI	Initial percentage of positive biopsies	Time to relapse	Tumor location at relapse	Percentage of positive biopsies at relapse
Patient 1	Left apex	None	8%	18 months	Left lobe (MRI)	NA
Patient 2	NA	Base and midgland	8%	36 months	Left apex (MRI)	NA
Patient 3	Right midgland	Midgland	8%	43 months	Left base and seminal vesicle (prostatectomy)	NA
Patient 4	Left and right midglands and bases	Midgland	16%	30 months	Apex (choline PET/CT)	NA
Patient 5	Left and right bases and apexes, left midgland	None	38%	91 months	All sextants, right seminal vesicle (biopsies)	72%
Patient 6	Left apex, right base	Base, midgland and apex	43%	44 months	Right lobe (MRI)	0%
Patient 7	Right base, left and right midglands and apexes	None	58%	54 months	Right base (biopsies)	12%

Abbreviations: *NA* Not available

The results of the univariate analysis are presented in Table 3. None of the studied covariates (percentage of positive biopsies, location of positive biopsies, pre-treatment PSA serum level, Gleason score, clinical and radiological tumoral stage, clinical risk group, radiological risk group, adjunction of neo-adjuvant therapy and its duration, D90 and V100) was found to be significantly associated with the progression-free survival. There was a trend towards an association with progression-free survival for the clinical risk group (HR = 1.73 [0.99–3.03], *p* = 0.055), and for the Gleason score, when studied separating Gleason scores < 7 and ≥ 7 (HR = 2.42 [0.99–5.93], *p* = 0.053). We also noted that there were more patients with Gleason scores ≥7 among patients who progressed than among patients who did not progress (40% vs 19%, *p* = 0.042).

**Table 3:** Results of the univariate analysis

	HR	95%CI	*p*-value
Pre-treatment PSA	1.09	0.92–1.29	0.341
Clinical tumor stage	1.14	0.48–2.71	0.765
Radiological tumor stage	1.12	0.71–1.75	0.627
Gleason score	1.71	0.87–3.34	0.115
Gleason score (< or ≥ 7)	2.42	0.98–5.93	0.053
Clinical risk group	1.73	0.98–3.03	0.055
Radiological risk group	1.17	0.78–1.75	0.441
Percentage of positive biopsies	1.02	0.99–1.05	0.191
Percentage of positive biopsies (≤ or > 50%)	2.34	0.53–10.15	0.257
Neo-adjuvant hormone therapy	1.57	0.64–3.87	0.323
Duration of hormone therapy	1.01	0.84–1.22	0.887
Apex involvement	1.16	0.44–3.06	0.758
Midgland involvement	0.83	0.31–2.19	0.716
Base involvement	1.26	0.47–3.41	0.6491
D90	1.04	0.92–1.16	0.5592
V100	2.67	0.01–1055.7	0.7479

Among the 20 patients who experienced a progression, the different rescue treatment options were: androgen deprivation therapy (total androgen blockade) for 11 patients, prostatectomy for 2 patients, and stereotactic external beam radiation therapy for 2 patients (one on the prostate, and one on a lombo-aortic lymphadenopathy). The information was missing for five patients.

## Discussion

Low-dose rate prostate brachytherapy is a very efficient treatment for localized prostate cancer, when patients are well selected.

Our results are consistent with other studies, with 5 year progression-free survival ranging from 85 to 97% for low risk prostate cancers [3, 7–10, 28, 35–38], and 75 to 94% for intermediate risk prostate cancers [7–10, 36, 37].

The oncological outcome is also comparable with other treatment modalities that can be proposed to patients with low to intermediate risk prostate cancers. The 5-year progression free survival rates for these patients range from 85 to 97% for radical prostatectomy [28, 35, 39], from 80 to 94.5% for high-dose external beam radiation therapy (74 Gy or more) [3, 39–42], and from 60 to 75% for active surveillance [43–45].

Rescue treatments with a curative intent after prostate brachytherapy are technically difficult. It is therefore very important to adequately select the patients who will benefit from brachytherapy.

Classical factors, such as PSA serum level, Gleason score, and tumor stage, which are taken into account in the risk groups according to D’Amico’s classification, are used to select patients, as they have been proven to be associated with progression-free survival [1, 7, 8, 10, 19, 46–48]. Other factors, such as tumor location and tumor burden, might be associated with progression-free-survival, and are frequently considered when selecting patients.

In our cohort, we were unable to identify a significant relationship between the percentage of positive biopsies and progression-free-survival, probably due to a lack of power but also possibly because of the difficulties we have in evaluating the tumor burden. This relationship between percentage of positive biopsies and biochemical outcome has been proven for prostate cancers treated by prostatectomy, or by external-beam radiation therapy [15]. It is not sure however if these results can be applied to brachytherapy. The tumor burden (expressed as percentage of positive biopsies, surface area positive for cancer, or number of positive cores) has been shown to be associated with extra-prostatic extension [49–54]. Compared to prostatectomy, brachytherapy enables an efficient treatment of the periprostatic tissues [55]. Concerning external-beam radiation therapy, the dose which can be delivered on the prostate might be insufficient in case of a high tumor burden, but this limitation could be overcome with brachytherapy, because it enables an important dose escalation on the prostate [56–58]. These two factors might be responsible for a potential lack of influence of tumor burden on biochemical outcome after brachytherapy.

The association between the local tumor burden and the biochemical outcome has already been studied for patients treated with brachytherapy in a few retrospective studies, but the results are discordant (Table 4) [16–20, 57–61]. Rossi et al. [17] found that patients with more than 50% of positive biopsies had a biochemical progression-free survival of 95%, whereas patients with less than 50% of positive biopsies had a biochemical progression-free survival of only 63% (*p* < 0.0001). Guzzo et al. [18] also noted that the percentage of positive biopsies was independently associated with the biochemical progression-free survival (when analyzed in a categorical fashion, with a threshold of 27%, which was the median percentage of positive biopsies in their cohort). Grann et al. [16] found no association between the percentage of positive biopsies and biochemical progression-free survival, they did however find a tendency for an association between tumor length and biochemical progression-free survival (*p* = 0.15). Merrick et al. [57] found an association between percentage of positive biopsies and biochemical progression-free survival, but this association was not significant when stratifying patients by risk groups. On the other hand, Hill et al. [60], and Martell et al. [61] found no association between percentage of positive biopsies and biochemical progression-free survival.

**Table 4:** Relationship between tumor burden and outcome in the literature

	Period of recruitment	Number of patients	Median number of biopsy cores	Median follow-up	Outcome
Grann et al., 1998 [16]	1988–1994	103	NA	3 years	5-year bPFS = LT < 10 mm: 74% LT > 10 mm: 36% (*p* = 0.011, univariate / *p* = 0.15 multivariate)
Merrick et al., 2002 [57]	1995–1999	262 (111 with exclusive brachytherapy)	6 (2–15)	38.6 months	5-year bPFS = 92.5% PPB < 34%: 96.6% PPB 34–50%: 92.9% PPB > 50%: 84.8% (*p* = 0.017) PPB not associated with bPFS within each risk group.
Merrick et al., 2004 [58]	1995–2001	413 (197 with exclusive brachytherapy)	NA	52 months	7-year bPFS = PPB < 34%: 99.4% PPB 34–50%: 94.3% PPB > 50%: 89.2% (*p* = 0.002)
Rossi et al., 2006 [17]	1997–1999	108	12	61 months	5-year bPFS = 87% PPB < 50%: 95% PPB > 50%: 63% (*p* < 0.0001)
Guzzo et al., 2008 [18]	1992–2002	245	6.8 (1–18)	52.8 months	5-year bPFS: 82% PPB < 27%: 89.1% PPB > 27%: 73.8% (*p* = 0.011)
Merrick et al., 2008 [59]	1995–2004	145		5.8 years	9-year bPFS = 97.1% PPB = predictor of bPFS in multivariate analysis.
Potters et al., 2008 [19]	1992–2000	1449	NA	82 months	12-year bPFS = 78% PPB independently associated with bPFS in the multivariate analysis (*p* = 0.037).
Taira et al., 2011 [20]	1995–2006	1656 (831 with exclusive brachytherapy)	NA	7 years	12-year bPFS = 95.6% PPB > 34%: 98% PPB 34–50%: 93% PPB > 50%: 91.5% (*p* < 0.001)
Hill et al., 2015 [60]	1998–2012	846	12	5.59 years	Biochemical failures: 62 (7.3%) Failures: PPB = 32.8% Non failures: PPB = 34.9% NS
Martell et al., 2017 [61]	2003–2013	2608	NA	4,7 years	Estimated 7-year-bPFS = 93% PPB not associated with bPFS.
Present study	2006–2013	282	12 (5–32)	61 months	Biochemical failures: 20 (7.1%) Failures: PPB =17% Non failures: PPB = 17% NS

Abbreviations: *bPFS* biochemical Progression Free Survival, *PPB* Percentage of Positive Biopsies, *LT* length of tumor, *NS* Non significant

The adequate way to assess the tumor burden and its association with biochemical outcome is not well defined: either tumoral length, or percentage of positive biopsies, with different possible statistical analyses (linear, or categorical, with different possible thresholds [16–18, 20, 57, 58]. Furthermore, one of the main issues in evaluating the local tumor burden with prostate biopsies is that there is an important sampling bias [62]. It seems that the more prostate biopsies are performed, the more accurate the percentage of prostate biopsies will be as a prognosis factor [18]. The number of biopsy cores performed has been shown to be an independent prognosis factor for patients treated with brachytherapy [63]. The realization of targeted prostate biopsies, becoming more frequent with the generalization of pre-biopsy procedure prostate MRI, also distorts the evaluation of tumor burden. These factors might contribute to explain why we did not find a significant association between percentage of positive biopsies and progression-free-survival.

In daily practice, the percentage of positive biopsies is a factor that clinicians consider in their therapeutic choice. A survey made in 2007 in the USA found that radiation oncologists were influenced and more prone to treating patients with external beam radiation therapy, when positive biopsies increased from 30 to 50% [64].

Concerning tumor location, only few authors have studied this aspect in patients treated with brachytherapy (Table 5). Hill et al. [60] found no significant association between base involvement and progression-free survival, but only a trend with a progression-free survival at ten years of 88.2% for patients with base involvement versus 92% for patients without base involvement. In a non-comparative retrospective study, Samuelian et al. [65] found that patients with base involvement treated with exclusive brachytherapy had a good disease control, with a progression-free survival of 93.5% at 10 years.

**Table 5:** Relationship between tumor location and outcome in the literature

	Period of recruitment	Number of patients	Median number of biopsy cores	Tumor location	Median follow-up	Outcome
Samuelian et al., 2011 [65]	1998–2006	52	7	BI: 52	89 months	10-year bPFS = 93.5%
Hill et al., 2015 [60]	1998–2012	846	12	BI: 528 MI: 578 AI: 560 Non BI: 318	5.59 years	Biochemical failures: 62 (7.3%) BI: 42/62 (67.7%) No BI: 20/62 (32.3%) (*p* = 0.17)
Present study	2006–2013	282	12	BI: 145 MI: 149 AI: 131	61 months	Biochemical failures: 20 (7.1%) Failures: BI 11patients (55%) / MI 10 (50%) / AI 10 (50%) Non failures: BI 134 patients (51.1%)/ MI 139 (53.1%)/ AI 121 (46.2%) NS

Abbreviations: *BI* base involvement, *MI* midgland involvement, *AI* apex involvement

Yet, in prostate brachytherapy, the prostate base has been shown to be less well covered [22], with several technical limitations potentially explaining this underdosage. A correlation seems to exist between dosimetric quality and biochemical outcome [27, 66, 67], but this correlation might not be linear [68]. This could explain the good results observed even for cancers located at the prostate base, despite an underdosage of this location.

Furthermore, among low-risk prostate cancer, there seems to be very few tumors located in the anterior base of the prostate, which is not the case for intermediate risk prostate cancer [69]. These data might suggest that an underdosage of the prostate base would be of little consequence, at least for patients with low risk prostate cancer.

Our study did not show any association between tumor location and progression-free survival. Even though our study lacks the power to definitely conclude, it is a supplementary argument for safely proposing brachytherapy to patients with low to favorable-intermediate risk prostate cancer, regardless of the cancer location (except for technical contra-indications).

Even though we did not find any statistically significant associations with progression-free survival, we did however find two factors that presented a strong tendency towards an association with progression-free survival: the clinical risk group according to the D’Amico classification modified by Zumsteg, and the Gleason score (with a HR of 2.42 [0.99–5.93] for a Gleason score ≥ 7 vs < 7).

These results are consistent with previous studies which found an association of the Gleason score, the tumoral stage, the pre-treatment PSA serum level, or the risk group, with the progression-free survival. Several studies found an association, which is expected, between risk groups and progression-free survival [1, 7, 8, 19]. The PSA serum level, is also often found to be associated with progression-free survival [7, 8, 10, 19, 46, 47], but this association is most often described when comparing PSA values of more than 20 ng/mL (which would currently constitute a contra-indication for brachytherapy) with PSA values of 20 ng/mL or less. The Gleason score is also found to be independently associated with progression-free survival [10, 19, 48], as well as the tumoral stage, with worse outcomes described for stages T3 [8] or T2b and more [10, 46].

The strengths of our study are the important size of the cohort, and the non-selective recruitment of patients (consecutive patients treated between 2006 and 2013 were included). The monocentric character of our study also ensures that all patients received the same treatment according to the same protocol. However, our study has some limitations that should be considered. The low number of events (because of the low progression rates) does not grant us a significant amount of power to definitely conclude. The retrospective nature of our study, and the important number of patients who were excluded from our analysis (mainly because of unavailable pathology reports) are other limitations. Also, the five-year median follow-up was probably too short to account for a large number of events.

## Conclusions

Brachytherapy is an efficient treatment for localized prostate cancer when patients are selected with classical criteria (low to favorable intermediate risk prostate cancer). In this cohort of 282 patients, we did not find any significant association between either tumor location or percentage of positive biopsies, and progression-free-survival. We did, however, find a strong trend towards an association of both the clinical risk group (according to the D’Amico classification modified by Zumsteg) and the Gleason score, with progression-free survival. Although our study lacks the power to definitely conclude, these results support the continued use of brachytherapy, with no limitation regarding tumor location, for patients with low-risk and selected favorable intermediate risk prostate cancers (remaining cautious with these patients and not cumulating risk factors).

## Data Availability

The datasets used and/or analysed during the current study are available from the corresponding author on reasonable request.
